# Prognostic Value of the Inflammatory Burden Index (IBI) in Metastatic Urothelial Carcinoma Prior to First-Line Therapy

**DOI:** 10.3390/medicina62061027

**Published:** 2026-05-25

**Authors:** Irem Bilgetekin, Necla Demir, Emrah Eraslan, Zeynep Akdagcik, Ilknur Deliktas Onur, Ozturk Ates, Umut Demirci

**Affiliations:** 1 Department of Internal Medicine, Faculty of Medicine, Uskudar University, 34662 Istanbul, Turkey; 2 Department of Medical Oncology, Memorial Ankara Hospital, 06520 Ankara, Turkey; 3 Department of Medical Oncology, Dr. Abdurrahman Yurtaslan Ankara Oncology Training and Research Hospital, University of Health Sciences, 06200 Ankara, Turkey; 4 Department of Urology, Dr. Abdurrahman Yurtaslan Ankara Oncology Training and Research Hospital, University of Health Sciences, 06200 Ankara, Turkey

**Keywords:** inflammatory burden index, metastatic bladder cancer, prognostic biomarkers

## Abstract

*Background and Objectives*: The systemic inflammatory response is important in cancer prognosis and progression. The inflammatory burden index (IBI) provides information about both inflammation and the immune response. Urothelial carcinomas are immunogenic; therefore, it has been suggested that inflammatory indices may predict disease prognosis. The aim of this study was to investigate the effects of systemic inflammatory indices, particularly the inflammatory burden index, on disease progression and overall survival in patients with metastatic urothelial cancer (affecting the bladder and upper urinary system) before first-line treatment and to demonstrate their prognostic importance. *Materials and Methods*: Within the scope of the study, the medical records of 130 patients who received systemic treatment for metastatic urothelial carcinoma at the medical oncology clinic were retrospectively reviewed. Receiver operating characteristic (ROC) curve analysis was performed to determine the optimal threshold values for IBI. Survival rates were calculated using the Kaplan–Meier method, and survival differences between groups were compared with the log-rank test. Univariate and multivariate analyses were performed using the Cox proportional hazards regression model to evaluate prognostic factors. *Results*: A total of 130 patients were included in the study. The median age was 64.9 years (IQR: 57.2–70.5). The primary tumor location was the bladder in 84.6% of patients, while the remaining 15.4% originated from the ureter and renal pelvis. In first-line systemic treatment, patients received a median of 4 cycles (IQR: 3–6). The median number of total treatment lines administered for metastatic disease was 1 (IQR: 1–2). In progression-free survival (PFS) analyses, the median PFS was 9.20 (95% CI 6.55–11.85) months in the IBI-low group (*n* = 47) and 5.82 (95% CI 4.56–7.07) months in the IBI-high group (*n* = 83) (*p* < 0.001). The median OS was calculated to be 18.96 (95% CI 16.61–21.30) months in the IBI-low group (*n* = 47), while it was found to be 9.50 (95% CI 7.70–11.29) months in the IBI-high group (*n* = 83) (*p* < 0.001). In multivariate analysis, high IBI and the presence of brain metastasis were found to be associated with the risk of progression. In terms of overall survival, the presence of brain metastasis, the presence of visceral metastasis, ECOG PS status, receipt of maintenance therapy, LMR, and the IBI score showed statistically significant prognostic effects. *Conclusions*: In metastatic urothelial carcinoma, the IBI was identified as an independent prognostic factor associated with progression-free and overall survival. These findings suggest that the IBI may have potential utility as a prognostic biomarker; however, larger, multicenter, and prospective studies are required to further validate its clinical applicability.

## 1. Introduction

Urothelial cancers are immunogenic and generally associated with inflammatory processes. The incidence of metastatic urothelial cancer increases with age. Most patients diagnosed are aged 65 years and older, and carcinogens such as smoking and chemicals are involved in its etiology. Chronic inflammation (chronic irritation or infections) plays an important role in the development of the disease [1]. Although the stage of the disease is an important factor determining survival, inflammatory markers that are inexpensive to evaluate and effective for treatment decision-making and determining disease prognosis are useful. Systemic inflammation is an important feature of the tumor microenvironment and plays a vital role in disease progression and prognosis in patients with tumors [2]. The systemic inflammatory response is an important biological process in terms of prognosis and cancer progression [3,4].

Hematological inflammatory markers such as neutrophils, lymphocytes, platelets, and C-reactive protein (CRP) can effectively reflect the systemic inflammatory status of tumors [5,6]. Indices showing the systemic inflammatory response, such as the CRP-to-albumin ratio (CAR), systemic immune–inflammation index (SII), platelet-to-lymphocyte ratio (PLR), lymphocyte-to-monocyte ratio (LMR), and neutrophil-to-lymphocyte ratio (NLR), are used. These indices have prognostic significance [7,8,9,10].

The recently developed inflammatory burden index (IBI) is calculated as CRP (C-reactive protein) × NLR (neutrophil/lymphocyte ratio). It is a hematological index developed to evaluate the inflammatory status and survival of cancer patients [11] and has been validated as an effective prognostic marker in various malignancies such as gastric cancer and lung cancer. In one study, inflammatory biomarkers were investigated as potential determinants of prognosis in patients with gastric cancer. According to the results of this study, C-reactive protein (CRP), the neutrophil-to-lymphocyte ratio (NLR), the platelet-to-lymphocyte ratio (PLR), the Glasgow prognostic score (GPS), and the systemic immune–inflammation index (SII) were found to be associated with clinical outcomes and may be beneficial in determining prognosis [12,13,14,15,16,17,18].

The inflammatory burden index (IBI) has been validated as an effective prognostic marker in many malignancies [19,20,21,22]. However, its role in metastatic urothelial cancers has not been clearly demonstrated. We believe that demonstrating the relationship between pre-treatment IBI values and prognosis and survival in metastatic urothelial cancers in this study will contribute meaningfully to the literature.

## 2. Materials and Methods

### 2.1. Study Design and Patient Population

Within the scope of this study, the medical records of 130 patients who received systemic treatment for metastatic urothelial cancer at the medical oncology clinic were retrospectively reviewed from patient files and the electronic record system. The patients included in the study had received at least one line of treatment in the metastatic stage. The following patients were included in the study: those aged ≥18 years with a histologically confirmed diagnosis of urothelial carcinoma involving the bladder, ureter, or renal pelvis, who had metastatic disease and received first-line systemic treatment for metastatic urothelial carcinoma. Additionally, only patients with available baseline complete blood count and biochemical parameters prior to initiation of first-line treatment, as well as accessible clinical, pathological, treatment, and survival data in their medical records, were included. The following patients were excluded from the study: those with missing baseline laboratory data required to calculate inflammatory indices, including IBI; patients with chronic inflammatory disease, active infection, and chronic steroid/immunosuppressive drug use at baseline that could affect inflammatory markers.

Our study was approved by the ethics committee (research code no: 2025-07/14; date: 24 July 2025).

### 2.2. Data Collection and Clinical Variables

The patients’ age, sex, ECOG-PS (Eastern Cooperative Oncology Group—Performance Status), smoking status, presence of comorbid disease, tumor location, tumor grade, receipt of neoadjuvant therapy, operation status, recurrence status, metastatic site, CRP, neutrophil, monocyte, lymphocyte, and platelet values before first-line treatment in the metastatic setting, after first-line treatment was received, and progression status were recorded in the database through the electronic medical record system. Disease progression was defined according to RECIST 1.1 criteria as at least a 20% increase in the sum of diameters of target lesions (with an absolute increase of ≥5 mm), the development of new lesions, or unequivocal progression of non-target lesions [23].

### 2.3. Definition of Inflammatory Indices

The patients’ IBI score before first-line treatment was calculated as follows: inflammatory burden index (IBI) = C-reactive protein (mg/L) × neutrophil (×10^3^/µL)/lymphocyte (×10^3^/µL) [11,19,20,21,22,23,24].

• The hematological indices consider leukocytes, neutrophils, lymphocytes, monocytes, C-reactive protein, platelets, hemoglobin, and albumin.
• Hemogram parameters (neutrophil, lymphocyte, and platelet) and CRP level before first-line treatment were analyzed.
• IBI was calculated using the CRP × NLR formula.
• Other inflammatory and nutritional indices, such as the PLR (platelet/lymphocyte ratio) and PNI (Prognostic Nutritional Index), were also calculated.

Systemic inflammation-based indices were calculated [7,8,9,10].

Neutrophil-to-lymphocyte ratio (NLR) = NEU/LYM;

Platelet-to-lymphocyte ratio (PLR) = PLT/LYM;

Lymphocyte-to-monocyte ratio (LMR) = LYM/MON;

C-reactive protein-to-albumin ratio (CAR) = CRP/ALB;

C-reactive protein/lymphocyte ratio (CLR);

Inflammatory burden index (IBI) = CRP × NLR;

Pan immune inflammation value (PIV) = NEU × PLT × MON/LYM.

### 2.4. Statistical Analysis

All statistical evaluations were performed using the SPSS software (SPSS for Windows, version 25.0; SPSS Inc., Chicago, IL, USA). Non-parametric continuous variables are expressed as medians and interquartile range (IQRs), while categorical variables are expressed as frequencies and percentages. The Mann–Whitney U test was preferred for the comparison of non-parametric continuous data between groups. In the analysis of categorical variables, the Pearson chi-square test and, when necessary, Fisher’s exact test were used.

Receiver operating characteristic (ROC) curve analysis was performed to determine the optimal threshold values for IBI. Survival rates were calculated using the Kaplan–Meier method, and survival differences between groups were compared using the log-rank test. Univariate and multivariate analyses were performed using the Cox proportional hazards regression model to evaluate prognostic factors. Variables found to be statistically significant in univariate analyses (*p* < 0.05) were included in the multivariate model. All analyses were performed as two-sided, and a *p*-value <0.05 was accepted as the threshold for statistical significance. No formal adjustment for multiple testing was applied, as the analyses were exploratory in nature.

## 3. Results

### 3.1. Patients and Baseline Characteristics

A total of 130 patients with metastatic urothelial cancer, with a median age of 64.9 years (IQR: 57.2–70.5), were included in this study. Most patients were male (85.4%), and 80.0% had a history of smoking. Among the accompanying comorbidities, hypertension was most frequently detected (26.9%), while diabetes mellitus (8.5%), coronary artery disease (10.8%), and COPD (7.7%) were observed at lower rates. The primary tumor location was the bladder in 84.6% of patients, while the remaining 15.4% of tumors originated from the ureter and renal pelvis. Regarding tumor grade, 95.4% were high-grade and 4.6% were low-grade urothelial carcinoma. At the time of initial diagnosis, 52.3% of the patients were in the metastatic stage, 30.8% had muscle-invasive disease, and 16.9% were diagnosed with non-muscle-invasive bladder cancer (NMIBC). Most patients had stage 4B disease (76.0%). The most common metastatic sites were the lymph nodes (77.7%), bone (42.3%), and visceral organs (46.2%). The performance status of 82.3% of patients was ECOG 0–1.

In the first-line setting, the most commonly administered regimen was cisplatin plus gemcitabine in 54.6% of patients (*n* = 71), followed by carboplatin plus gemcitabine in 29.2% (*n* = 38). Paclitaxel was administered to 8.5% of patients (*n* = 11), while 4.6% (*n* = 6) received gemcitabine monotherapy. Nivolumab and MVAC were each used in 1.5% of patients (*n* = 2).

Maintenance therapy was administered to 19.2% of patients (*n* = 25), whereas 80.8% (*n* = 105) did not receive maintenance treatment. Among those who received maintenance therapy, the most frequently used agent was avelumab (84.0%, *n* = 21), followed by gemcitabine (16.0%, *n* = 4).

When laboratory parameters were evaluated, the median albumin level was 37.0 g/L (IQR: 33.0–40.0), the median CRP level was 21.0 mg/L (IQR: 10.4–45.0), the median neutrophil count was 5.3 × 10^3^/µL (IQR: 4.3–6.77), the lymphocyte count was 1.4 × 10^3^/µL (IQR: 1.1–1.9), and the platelet count was 286.5 × 10^3^/µL (IQR: 226.0–398.5). Among inflammation-based indices, the median NLR was calculated as 3.55 (IQR: 2.56–5.29), the PLR as 205.96 (IQR: 138.19–283.68), and the LMR as 2.69 (IQR: 1.96–3.52). The wide distribution of inflammatory biomarkers such as CAR, CLR, IBI, and PIV revealed that the patient population represented a biologically heterogeneous and aggressive metastatic disease group. In particular, the high variation in IBI and PIV was noteworthy.

In first-line systemic treatment, the patients received a median of 4 cycles (IQR: 3–6). The median number of total treatment lines administered in metastatic disease was 1 (IQR: 1–2). In the limited group of patients who received maintenance therapy (*n* = 25), the median number of maintenance cycles was 7 (IQR: 4.5–13).

The baseline demographic, clinical, and laboratory characteristics of the patients are presented in Table 1.

### 3.2. ROC Analysis

**Figure 1:**
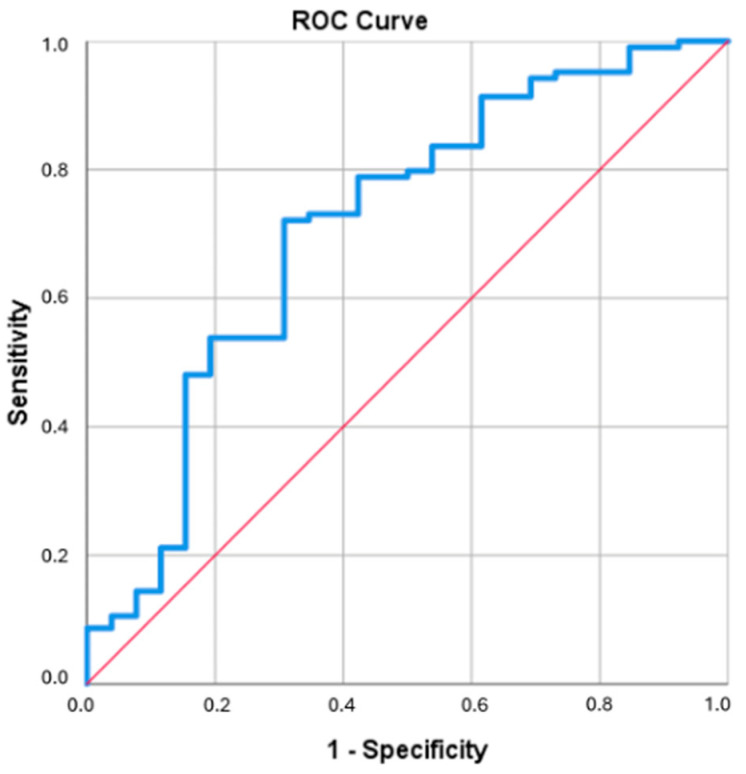
ROC analysis graph for the IBI score.

The discriminative power of the IBI score, one of the inflammation-based prognostic indices, for overall survival (OS) was evaluated using ROC analysis. As a result of the analysis, the area under the curve (AUC) for IBI was determined to be 0.711 (standard error: 0.061; 95% CI: 0.591–0.830; *p* = 0.001). The optimal cut-off point was determined to be 47.14. According to this cut-off value, patients were divided into two groups: IBI-low (<47.14) and IBI-high (≥47.14). The ROC analysis graph is shown in Figure 1.

### 3.3. Progression-Free Survival

**Figure 2:**
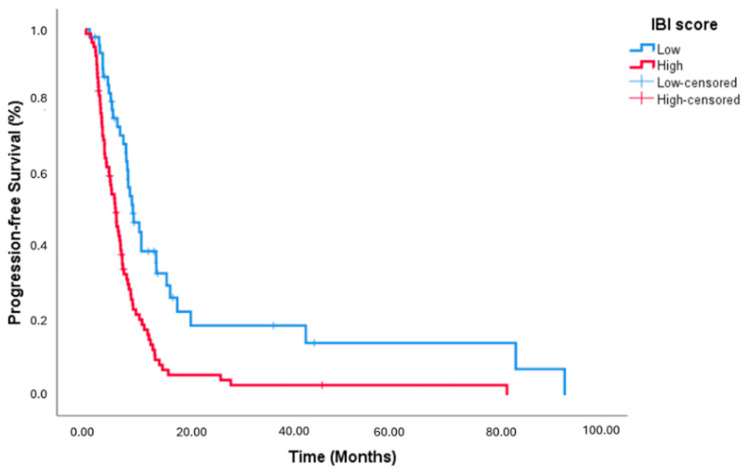
Comparative progression-free survival Kaplan–Meier curve according to IBI score.

In progression-free survival (PFS) analyses, the median PFS was calculated as 9.20 (95% CI 6.55–11.85) months in the IBI-low group (*n* = 47), whereas it was calculated as 5.82 (95% CI 4.56–7.07) months in the IBI-high group (*n* = 83) (*p* < 0.001). In the entire patient group (*n* = 130), the median PFS was 6.97 months (95% CI: 5.62–8.31). Kaplan–Meier survival curves demonstrated a significantly shorter PFS in the IBI-high group compared to the IBI-low group (Figure 2).

Clinical and biochemical variables that may influence progression-free survival were evaluated using univariate Cox regression analysis. In this analysis, the hazard ratio (HR) was calculated as 4.590 (95% CI: 1.651–12.761) for the presence of brain metastasis, 1.814 (95% CI: 1.125–2.926) for ECOG performance status (2 vs. 0–1), 0.422 (95% CI: 0.243–0.732) for maintenance therapy, 0.947 (95% CI: 0.912–0.983) for PD-L1, 1.123 (95% CI: 1.052–1.200) for NLR, 1.001 (95% CI: 1.000–1.001) for the PLR, 0.817 (95% CI: 0.717–0.931) for the LMR, 1.002 (95% CI: 1.001–1.003) for the IBI, and 1.000 (95% CI: 1.000–1.000) for the PIV.

Variables with *p* < 0.05 in the univariate analysis were included in the multivariate Cox regression model. As a result of the multivariate analysis, HR was found to be 3.409 (95% CI: 1.154–10.074) for brain metastasis, 0.705 (95% CI: 0.381–1.305) for maintenance therapy, 0.964 (95% CI: 0.930–1.000) for PD-L1, 0.890 (95% CI: 0.765–1.036) for the NLR, 1.001 (95% CI: 0.999–1.003) for the PLR, 0.879 (95% CI: 0.760–1.016) for the LMR, 1.001 (95% CI: 1.000–1.003) for the IBI, and 1.000 (95% CI: 1.000–1.000) for the PIV.

The results of the univariate and multivariate Cox regression analyses performed for factors that may predict progression-free survival (PFS) are presented in Table 2.

### 3.4. Overall Survival (OS)

**Figure 3:**
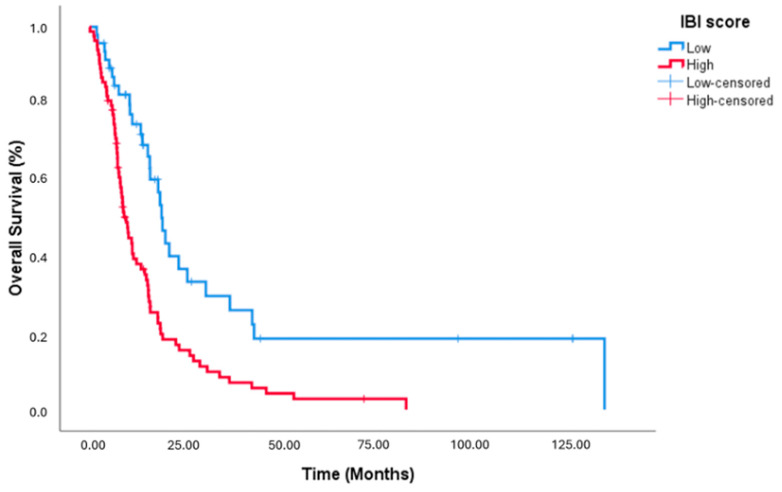
Comparative overall survival Kaplan–Meier curve according to IBI score.

The median OS was calculated as 18.96 (95% CI 16.61–21.30) months in the IBI-low group (*n* = 47), while it was found to be 9.50 (95% CI 7.70–11.29) months in the IBI-high group (*n* = 83) (*p* < 0.001). In the entire cohort (*n* = 130), the median OS duration was 13.31 months (95% CI: 9.89–16.72). Kaplan–Meier survival curves demonstrated significantly shorter OS in the IBI-high group compared to the IBI-low group (Figure 3).

In the univariate Cox regression analysis performed for overall survival, the presence of brain metastasis had an HR of 4.465 (95% CI: 1.790–11.138), the presence of visceral metastasis an HR of 1.566 (95% CI: 1.055–2.326), ECOG performance status (2 vs. 0–1) an HR of 2.379 (95% CI: 1.473–3.844), maintenance therapy an HR of 0.369 (95% CI: 0.185–0.736), the NLR an HR of 1.121 (95% CI: 1.050–1.197), the LMR an HR of 0.838 (95% CI: 0.734–0.958), the IBI an HR of 1.002 (95% CI: 1.001–1.003), and the PIV an HR of 1.000 (95% CI: 1.000–1.000).

In the multivariate Cox regression model created with variables that were significant in the univariate analysis (*p* < 0.05), the HR was calculated as 3.310 (95% CI: 1.263–8.676) for brain metastasis, 1.673 (95% CI: 1.090–2.569) for visceral metastasis, 2.241 (95% CI: 1.320–3.803) for ECOG performance status, 0.429 (95% CI: 0.208–0.884) for maintenance therapy, 0.843 (95% CI: 0.723–0.984) for the LMR, 1.002 (95% CI: 1.001–1.003) for the IBI, and 1.000 (95% CI: 1.000–1.000) for the PIV.

The results of the univariate and multivariate Cox regression analyses performed for factors that may predict overall survival (OS) are presented in Table 3.

### 3.5. Comparison of Groups According to IBI Score

According to the IBI score, 47 patients were classified into the low-IBI group and 83 patients into the high-IBI group. The gender distribution was 21.3% female (*n* = 10) and 78.7% male (*n* = 37) in the low-IBI group, and 10.8% female (*n* = 9) and 89.2% male (*n* = 74) in the high-IBI group (*p* = 0.106). At the time of diagnosis, metastatic disease was present in 51.1% (*n* = 24) of patients in the low-IBI group and 53.0% (*n* = 44) in the high-IBI group (*p* = 0.831), while non-metastatic disease was observed in 48.9% (*n* = 23) and 47.0% (*n* = 39), respectively. Bone metastases were observed in 34.0% (*n* = 16) of patients in the low-IBI group and 47.0% (*n* = 39) in the high-IBI group; visceral metastases in 48.9% (*n* = 23) and 44.6% (*n* = 37); and brain metastases in 6.4% (*n* = 3) and 2.4% (*n* = 2), respectively (*p* = 0.151, *p* = 0.632, and *p* = 0.258). An ECOG performance status score of 2 was present in 12.8% (*n* = 6) of the low-IBI group and 20.5% (*n* = 17) of the high-IBI group (*p* = 0.268). Maintenance therapy was administered to 34.0% (*n* = 16) of patients in the low-IBI group and 10.8% (*n* = 9) in the high-IBI group, demonstrating a statistically significant difference between groups (*p* = 0.001). For the low- and high-IBI groups, respectively, the mean number of treatment lines administered for metastatic disease was 1.62 ± 0.709 (range: 1–3) and 1.52 ± 0.705 (range: 1–3) (*p* = 0.373). The mean number of first-line treatment cycles was 4.98 ± 1.700 (range: 1–9) and 4.37 ± 1.806 (range: 1–9) (*p* = 0.055), while the mean number of maintenance therapy cycles was 10.31 ± 5.952 (range: 2–22) and 7.44 ± 5.388 (range: 2–19) (*p* = 0.181) (Table 4).

## 4. Discussion

In this study, we compared the prognostic effects of inflammatory indices before first-line treatment in metastatic urothelial cancer and found that the IBI was most closely associated with prognosis and that patients with a high IBI had significantly worse outcomes in terms of both PFS and OS, when compared to patients with a low IBI. These results are important for determining prognosis before treatment in metastatic urothelial cancers. Uncontrolled inflammation is closely associated with tumor formation, development, invasion, and metastasis [25,26]. As an effective defense against tumor cells, lymphocytes play a role in immune regulation within the tumor microenvironment [27].

The IBI includes three main parameters (CRP, neutrophils, and lymphocytes). While an increase in serum CRP is considered the most important clinical parameter of acute inflammation, neutrophils and lymphocytes are also highly important in the immune response [28,29,30]. Persistently elevated CRP levels generally predict poor prognosis and indicate metastasis in cancer patients [31,32]. In the evaluation of prognosis in patients with NSCLC, the IBI has been shown to be advantageous in measuring the balance between acute and immune inflammation by combining CRP, neutrophil, and lymphocyte measurements [26]. Previous studies have evaluated the association between the IBI and survival in cancer. Notably, Xie H. et al. demonstrated that increased inflammatory burden was independently associated with adverse survival outcomes in different cancer types [11]. In a study conducted by Ding et al. (2023) involving patients with locally advanced gastric cancer, a high IBI was associated with lower 5-year OS and DFS compared to a low IBI (OS: 70% vs. 79.1%; DFS: 50% vs. 74.4) [19].

As a result of our study, among inflammatory indices, the IBI score was shown to have prognostic importance for patients with metastatic urothelial cancer and was found to be an independent predictive factor for disease progression and overall survival. While the median OS was determined to be 18.96 (95% CI 16.61–21.30) months in the IBI-low group (*n* = 47), it was found to be 9.50 (95% CI 7.70–11.29) months in the IBI-high group (*n* = 83) (*p* < 0.001). Some previous studies reported that the IBI is a strong prognostic factor in lung, esophageal, and colorectal cancers, and high IBI values have been associated with poor survival [32,33,34]. The first evaluation of the IBI was conducted in a prospective multicenter study involving 6359 cancer patients, and their results indicated that the IBI was an independent high-risk variable associated with short-term outcomes, nutritional status, and functional status. In terms of survival rate, patients with low IBI scores had better outcomes (45.7% vs. 69.1%; *p* < 0.001) [11]. Xhi et al. found that lung cancer patients with high IBI scores had significantly worse survival after surgical resection compared to those with low IBI scores (35.46% vs. 57.22%; *p* < 0.001) [21]. Ding et al. obtained similar results in gastric cancer [19].

To enhance clinical interpretability, the IBI cutoff identified in our study (IBI = 47.14) can be directly translated into routinely measured laboratory parameters. The IBI is derived from commonly available inflammatory markers, including CRP, neutrophil count, and lymphocyte count, making it easily applicable in daily clinical practice without additional testing.

For example, a patient with elevated CRP and neutrophil levels accompanied by relative lymphopenia would yield an IBI value above the cutoff, corresponding to the high-IBI group, whereas patients with lower CRP and balanced leukocyte counts would typically fall into the low-IBI group.

Since CRP is important in the evaluation of the IBI score, CRP monitoring is important. In our study, the presence of brain metastasis, PD-L1 status and the IBI score were found to be independent prognostic factors for progression-free survival. Riedl et al. found that the effectiveness of PD-L1 in patients with advanced non-small cell lung cancer was negatively correlated with pre-treatment CRP levels [35]. Other systemic inflammatory indices were not found to be significant in predicting progression-free survival in metastatic urothelial cancer.

Chemotherapy reduces the inflammatory response, and as the tumor burden decreases with chemotherapy, systemic inflammation is also suppressed. In a gastric cancer study in patients receiving neoadjuvant immunotherapy, tumor regression was worse in the high-IBI group, postoperative complications were more frequent, and the overall survival rate was lower compared to the low-IBI group [36]. In another prospective study in patients with non-small cell lung cancer, a high IBI score was found to be associated with cachexia, risk of death, and 90-day complications [21]. Similarly, in our study, overall survival was shorter in patients with a high IBI compared to those with a low IBI. Although parameters such as neutrophils, lymphocytes, and CRP have previously been used, as also shown in our study, the IBI score demonstrated prognostic significance for both overall survival and progression-free survival.

The therapeutic landscape of metastatic urothelial carcinoma has undergone a fundamental shift with the approval of enfortumab vedotin plus pembrolizumab as first-line standard of care, demonstrating a median overall survival of 31.5 months versus 16.1 months with platinum-based chemotherapy in the phase III EV-302 trial [37]. Beyond established agents, next-generation antibody–drug conjugates, targeted therapies, and innovative intravesical approaches continue to expand treatment options across disease stages [38]. In this evolving context, pre-treatment inflammatory indices such as the IBI may serve a complementary role in risk stratification, particularly given that systemic inflammation can modulate the tumor microenvironment and influence immunotherapy efficacy through gut microbiota–immune interactions, whereby commensal bacteria regulate the effector functions of immune cells and thereby affect responses to both chemotherapy and immune checkpoint inhibitors [39].

However, these findings should be interpreted with caution. Although IBI remained statistically significant in the multivariate analysis, we did not directly compare its prognostic performance with other inflammatory markers. For this reason, our results do not indicate that IBI is superior but rather suggest that it may have value as an independent prognostic factor. Overall, these findings should be seen as preliminary and need to be confirmed in larger, prospective, and comparative studies.

### Strengths and Limitations of the Study

Given the retrospective design of our study, some degree of heterogeneity in treatment regimens, including differences in treatment lines and cycle numbers, is unavoidable. However, the IBI was calculated before the initiation of first-line therapy, thereby reflecting the baseline inflammatory status of the disease rather than treatment-related changes. In addition, patients with conditions that could potentially influence inflammatory markers, such as active infections, chronic inflammatory diseases, or the use of steroids or other immunosuppressive agents, were excluded at baseline.

Another limitation is that the IBI was assessed only at a single pre-treatment time point. Systemic inflammation is a dynamic process that may evolve during the course of treatment, and therefore, changes in IBI over time were not captured in this study. Longitudinal assessment of IBI may provide additional insight into treatment response and disease progression, and future prospective studies incorporating serial measurements are needed to clarify its potential role as a monitoring biomarker.

Although variability in treatment may still have an impact on survival outcomes, the consistent association observed between higher pre-treatment IBI levels and poorer prognosis suggests that this index is more likely to reflect underlying tumor biology. That said, residual confounding cannot be entirely ruled out, and prospective studies in more homogeneous patient populations would be valuable to confirm these findings. It is also important to note that our cohort predominantly reflects a chemotherapy-based treatment era, as newer combinations such as enfortumab vedotin plus pembrolizumab were not widely available during the study period. This may limit the applicability of our results to current treatment settings.

One of the main strengths of our study is that, to our knowledge, it is among the first to specifically investigate the prognostic role of the IBI in metastatic urothelial carcinoma, thereby addressing an existing gap in the literature. In addition, the use of widely accessible and cost-effective laboratory parameters, together with a well-defined real-world cohort, increases the potential clinical relevance of our findings. While further prospective and longitudinal studies are needed, our results provide an initial indication that IBI may serve as a useful prognostic marker in this patient population.

## 5. Conclusions

In conclusion, the IBI appears to be a useful prognostic biomarker in metastatic urothelial carcinoma. Since it is based on simple, inexpensive, and routinely available laboratory parameters, it could be easily applied in clinical practice.

Larger, multicenter studies are needed to better define the role of the IBI in prognostic value and clinical decision-making in metastatic urothelial carcinoma.

## Figures and Tables

**Table 1:** Demographic, clinical, and laboratory characteristics of the patients.

Variable	Value
Age (years)	64.9 (57.2–70.5)
Sex (male), n (%)	111 (85.4)
Sex (female), n (%)	19 (14.6)
Smoking history, n (%)	104 (80.0)
Presence of comorbidity, n (%)	35 (26.9)
Primary tumor location (bladder), n (%)	110 (84.6)
High-grade tumor, n (%)	124 (95.4)
Metastatic disease at diagnosis, n (%)	68 (52.3)
Metachronous metastasis	62 (47.7)
Stage 4B disease, n (%)	99 (76.0)
ECOG PS (0–1), n (%)	107 (82.3)
Albumin (g/L)	37.0 (33.0–40.0)
CRP (mg/L)	21.0 (10.4–45.0)
Neutrophil (×10^3^/µL)	5.3 (4.3–6.773)
Lymphocyte (×10^3^/µL)	1.4 (1.1–1.9)
Monocyte (×10^3^/µL)	0.54 (0.45–0.66)
Platelet (×10^3^/µL)	286.5 (226.0–398.5)
NLR	3.55 (2.56–5.29)
PLR	205.96 (138.19–283.68)
LMR	2.69 (1.96–3.52)
CAR	0.78 (0.32–1.67)
CLR	16.12 (7.95–40.64)
IBI	68.45 (32.40–191.89)
PIV	569.27 (310.21–1180.25)
Number of first-line treatment cycles	4 (3–6)
Number of metastatic treatment lines	1 (1–2)

Continuous variables are presented as median (interquartile range, IQR). ECOG PS: *Eastern Cooperative Oncology Group Performance Status;* CRP: *C-reactive protein*; NLR: *Neutrophil-to-Lymphocyte Ratio*; PLR: *Platelet-to-Lymphocyte Ratio*; LMR: *Lymphocyte-to-Monocyte Ratio*; CAR: *C-reactive protein-to-albumin ratio*; CLR: *C-reactive protein-to-lymphocyte ratio*; IBI: *Inflammatory burden index*; PIV: *Pan immune-inflammation value*.

**Table 2:** Univariate and multivariate Cox regression analysis for factors predicting progression-free survival (PFS).

Variable	Univariate		Multivariate	
	HR (95% CI)	*p*	HR (95% CI)	*p*
Age	0.977 (0.975–1.019)	0.763	–	
Sex	0.661 (0.395–1.507)	0.116	–	
Metastatic disease at diagnosis	1.045 (0.719–1.518)	0.819	–	
Type of metastatic disease (metachronous vs. de novo)	1.195 (0.818–1.745)	0.357	–	
Brain metastasis	4.590 (1.651–12.761)	0.003	3.409 (1.154–10.074)	0.027
Bone metastasis	0.970 (0.662–1.420)	0.875	–	
Visceral metastasis	1.290 (0.887–1.878)	0.183	–	
ECOG (2 vs. 0–1)	1.814 (1.125–2.926)	0.015	1.373 (0.794–2.374)	0.256
Neoadjuvant therapy	0.430 (0.106–1.749)	0.238	–	
Maintenance therapy	0.422 (0.243–0.732)	0.002	0.705 (0.381–1.305)	0.266
PD-L1	0.947 (0.912–0.983)	0.005	0.964 (0.930–1.000)	0.050
NLR	1.123 (1.052–1.200)	0.001	0.890 (0.765–1.036)	0.133
PLR	1.001 (1.000–1.001)	0.024	1.001 (0.999–1.003)	0.192
LMR	0.817 (0.717–0.931)	0.002	0.879 (0.760–1.016)	0.082
CAR	1.023 (0.951–1.099)	0.542	–	
CLR	1.001 (0.999–1.002)	0.549	–	
IBI	1.002 (1.001–1.003)	<0.001	1.001 (1.000–1.003)	0.006
PIV	1.000 (1.000–1.000)	0.001	1.000 (1.000–1.000)	0.162

ECOG: *Eastern Cooperative Oncology Group*; PD-L1: *Programmed Cell Death Ligand-1*; NLR: *Neutrophil-to-Lymphocyte Ratio*; PLR: *Platelet-to-Lymphocyte Ratio*; LMR: *Lymphocyte-to-Monocyte Ratio;* CAR: *C-reactive protein-to-albumin ratio*; CLR: *C-reactive protein-to-lymphocyte ratio*; IBI: *Inflammatory burden index*; PIV: *Pan immune-inflammation value*.

**Table 3:** Univariate and multivariate Cox regression analysis for factors predicting overall survival (OS).

Variable	Univariate		Multivariate	
	HR (95% CI)	*p*	HR (95% CI)	*p*
Age	1.008 (0.986–1.031)	0.463	–	
Sex	1.298 (0.738–2.283)	0.366	–	
Metastatic disease at diagnosis	0.956 (0.648–1.409)	0.820	–	
Type of metastatic disease (metachronous vs. de novo)	1.165 (0.784–1.731)	0.451	–	
Brain metastasis	4.465 (1.790–11.138)	0.001	3.310 (1.263–8.676)	0.015
Bone metastasis	0.776 (0.522–1.154)	0.211	–	
Visceral metastasis	1.566 (1.055–2.326)	0.026	1.673 (1.090–2.569)	0.019
ECOG (2 vs. 0–1)	2.379 (1.473–3.844)	<0.001	2.241 (1.320–3.803)	0.003
Neoadjuvant therapy	0.527 (0.130–2.145)	0.371	–	
Maintenance therapy	0.369 (0.185–0.736)	0.005	0.429 (0.208–0.884)	0.022
PD-L1	0.971 (0.941–1.003)	0.076	–	
NLR	1.121 (1.050–1.197)	0.001	0.900 (0.782–1.035)	0.141
LMR	0.838 (0.734–0.958)	0.009	0.843 (0.723–0.984)	0.030
CAR	1.029 (0.946–1.120)	0.506	–	
CLR	1.000 (0.998–1.003)	0.844	–	
IBI	1.002 (1.001–1.003)	<0.001	1.002 (1.001–1.003)	0.002
PIV	1.000 (1.000–1.000)	<0.001	1.000 (1.000–1.000)	0.035

ECOG: *Eastern Cooperative Oncology Group*; PD-L1: *Programmed Cell Death Ligand-1;* NLR: *Neutrophil-to-Lymphocyte Ratio*; PLR: *Platelet-to-Lymphocyte Ratio*; LMR: *Lymphocyte-to-Monocyte Ratio*; CAR: *C-reactive protein-to-albumin ratio*; CLR: *C-reactive protein-to-lymphocyte ratio*; IBI: *Inflammatory burden index*; PIV: *Pan immune-inflammation value*.

**Table 4:** Comparative evaluation of baseline clinical characteristics according to IBI score.

Variable	Low IBI (*n* = 47)	High IBI (*n* = 83)	*p* Value
Gender (female), *n* (%)	10 (21.3%)	9 (10.8%)	0.106
Metastatic disease, *n* (%)	24 (51.1%)	44 (53.0%)	0.831
Bone metastasis, *n* (%)	16 (34.0%)	39 (47.0%)	0.151
Visceral metastasis, *n* (%)	23 (48.9%)	37 (44.6%)	0.632
Brain metastasis, *n* (%)	3 (6.4%)	2 (2.4%)	0.258
ECOG PS = 2, *n* (%)	6 (12.8%)	17 (20.5%)	0.268
Maintenance therapy, *n* (%)	16 (34.0%)	9 (10.8%)	0.001
Number of metastatic treatment lines, mean ± SD (min–max)	1.62 ± 0.709 (1–3)	1.52 ± 0.705 (1–3)	0.373
Number of first-line cycles, mean ± SD (min–max)	4.98 ± 1.700 (1–9)	4.37 ± 1.806 (1–9)	0.055
Number of maintenance cycles, mean ± SD (min–max)	10.31 ± 5.952 (2–22)	7.44 ± 5.388 (2–19)	0.181

IBI: *Inflammatory Burden Index*; ECOG PS: *Eastern Cooperative Oncology Group Performance Status*; SD: *Standard Deviation*; min–max: *minimum–maximum values*.

## Data Availability

The data presented in this study are available on request from the corresponding author.

## Abbreviations

The following abbreviations are used in this manuscript:

AUC	Area Under Curve
CAR	C-Reactive Protein-to-Albumin Ratio
CI	Confidence Interval
CLR	C-Reactive Protein-to-Lymphocyte Ratio
COPD	Chronic Obstructive Pulmonary Disease
CRP	C-Reactive Protein
ECOG-PS	Eastern Cooperative Oncology Group-Performance Status
GPS	Glasgow Prognostic Score
HR	Hazard Ratio
IBI	Inflammatory Burden Index
IQR	Interquartile Range
LMR	Lymphocyte-to-Monocyte Ratio
NMIBC	Non-Muscle-Invasive Bladder Cancer
NLR	Neutrophil-to-Lymphocyte Ratio
OS	Overall Survival
PD-L1	Programmed Cell Death Ligand-1
PFS	Progression-Free Survival
PIV	Pan Immune-Inflammation Value
PLR	Platelet-to-Lymphocyte Ratio
PNI	Prognostic Nutritional Index
SII	Systemic immune-inflammation index
SPSS	Statistical Package for the Social Sciences
